# Changes in Nutritional Status and Musculoskeletal Health in a Geriatric Post-Fall Care Plan Setting

**DOI:** 10.3390/nu11071551

**Published:** 2019-07-09

**Authors:** Romy Conzade, Steven Phu, Sara Vogrin, Ebrahim Bani Hassan, Walter Sepúlveda-Loyola, Barbara Thorand, Gustavo Duque

**Affiliations:** 1 Australian Institute for Musculoskeletal Science (AIMSS), The University of Melbourne and Western Health, St. Albans, Victoria 3021, Australia; 2 Department of Medicine—Western Health, Melbourne Medical School, The University of Melbourne, St. Albans, Victoria 3021, Australia; 3 Institute of Epidemiology, Helmholtz Zentrum München, German Research Center for Environmental Health (GmbH), 85764 Neuherberg, Germany; 4 Department of Physiotherapy, Londrina State University (UEL) and University North of Paraná (UNOPAR), Londrina, Paraná 86041-120, Brazil

**Keywords:** malnutrition, MNA, nutrition, physical performance, bone turnover, sarcopenia, osteosarcopenia, falls, elderly, prospective

## Abstract

Understanding how changes in nutritional status influence musculoskeletal recovery after falling remains unclear. We explored associations between changes in nutritional status and musculoskeletal health in 106 community-dwelling older adults aged ≥65 years, who attended the Falls and Fractures Clinic at Sunshine Hospital in St Albans, Australia after falling. At baseline and after 6 months, individuals were assessed for Mini Nutritional Assessment (MNA^®^), grip strength, gait speed, Timed Up and Go (TUG) test, Short Physical Performance Battery (SPPB), and bone turnover marker levels. Associations were examined using multiple linear regression, adjusted for baseline covariates and post-fall care plans. Over 6 months, the prevalence of malnutrition or risk thereof decreased from 29% to 15% using MNA <24/30. Specifically, 20 individuals (19%) improved, 7 (7%) deteriorated, and 73 (69%) maintained nutritional status, including 65 (61%) who remained well-nourished and 8 (8%) who remained malnourished/at risk. A 1-point increase in MNA score over 6 months was associated with an increase of 0.20 points (95% confidence interval 0.10, 0.31, *p* < 0.001) in SPPB score. Improvement in nutritional status was associated with improvement in physical performance, providing a basis for interventional studies to ascertain causality and evaluate nutritional models of care for post-fall functional recovery in older adults.

## 1. Introduction

About a third of community-dwelling older adults aged ≥65 years in Western countries fall each year and the frequency of falls and fall-related injuries (fractures or head trauma) increase with age [1]. Falls increase the risk of hospitalization and nursing home admission, as well as morbidity and mortality [2]. Investigating modifiable risk factors of falls is a key priority area for healthcare systems, which strive to identify conditions that prevent falls.

Poor nutritional status has been considered an important modifiable risk factor for falls [3]. Compared to well-nourished older adults, risk of experiencing falls has been shown in a meta-analysis of prospective studies to be 45% higher in malnourished individuals or those at risk of malnutrition (*n* = 9510) [4], based on the validated Mini Nutritional Assessment (MNA^®^) tool [5]. In an interventional study by Swanenburg et al., the combination of a three-month calcium/vitamin D supplementation plus protein/exercise was associated with a 89% reduction in the rate of falls over 12 months compared to only calcium/vitamin D supplementation in older women aged ≥65 years with low mineral density (*n* = 20) [6]. Altogether, these findings suggest that nutritional status should be properly considered when assessing the risk of falls in community-dwelling older adults; yet it is currently not included in most fall risk screening tools [7].

Poor nutritional status can be a consequence of underlying comorbid conditions [8], which may increase the risk of falls due to clinical and adverse effects on cognitive, functional, and physical performance [9]. Poor nutritional status due to inadequate nutritional intake, especially of proteins, can also be detrimental for maintaining the integrity and function of skeletal muscle and bone [10,11,12], possibly increasing the risk for sarcopenia, osteoporosis or both [13,14,15,16]. Sarcopenia-associated risk of falling and increased bone vulnerability have a synergistic impact on falls and fractures occurrence [17,18]. The impact of nutritional status on the risk of falls can thus be explored through the pathway of musculoskeletal health as an important contributor to falls risk [19,20].

Evidence suggests that malnutrition based on MNA is able to predict musculoskeletal decline in various healthcare settings [21,22,23], but the relationship between nutritional changes and musculoskeletal outcomes remains under-researched [24], in particular among those who fall. Improving knowledge about how nutritional changes may influence relevant musculoskeletal outcomes might be important to effective targeting of multidisciplinary post-fall interventions for older adults living in the community. This study aimed to investigate changes in nutritional status in older adults with a history of falling using the validated MNA^®^, and to determine associations between changes in nutritional status and relevant musculoskeletal outcomes. We hypothesized that improvement in nutritional status is associated with greater musculoskeletal recovery.

## 2. Materials and Methods

### 2.1. Study Design and Individuals

This retrospective observational study examined associations between changes in nutritional status and musculoskeletal outcomes among community-dwelling older adults who attended the Falls and Fractures Clinic at the Australian Institute for Musculoskeletal Science (Western Health- Sunshine Hospital) in St Albans, VIC, Australia. A multidisciplinary team at the clinic, including a geriatrician, a fracture liaison nurse, an accredited exercise physiologist, and a bone densitometrist, provides comprehensive care for older adults with a history of more than two falls in the previous year, or a single fall with established gait and/or balance problem, and/or clinical or radiological risk of falls and/or fractures. We analyzed information from baseline attendance between October 2016 and December 2018 and from follow-up attendance after a median time of 6 months (interquartile range (Q1–Q3) 6–8 months). All measurements obtained were part of standard care practices at this health service. The Western Health Low Risk Ethics Panel approved the registration of the Falls and Fractures Clinic Databank (DB2017.13, date of approval 23 October 2018) and the research protocol of the present study (QA2018.90_48118, date of approval 5 December 2018). Participant consent was waived due to use of de-identified data collected as part of standard care at the clinic and due to the low risk nature of the study beyond the initial consent to attend the clinic.

### 2.2. Demographic and Clinical Measures

Demographic data was obtained from the patient medical record including age, gender, and residential location. Comprehensive clinical assessment was performed by the geriatrician and the nurse as part of routine care practices on clinic attendance including comorbidities, family history, fracture history, osteoporosis risk assessment (e.g., hormone replacement therapy, menopause age, smoking, alcohol), falls risk (e.g., hearing and visual deficit, altered elimination, impaired mobility), assessment for postural drop, and list of current medications. For the purpose of this study, a Charlson age-comorbidity index (CACI) was generated, with an index of ≥5 being suggestive of severe comorbidity [25]. The CACI calculation is explained in Supplementary Table S1. Polypharmacy was defined as use of ≥5 prescribed or regularly taken medications, including drugs and dietary supplements. Depression was screened using the Short Form Geriatric Depression Scale (GDS), with a score of ≥6/15 points considered as “suggestive of depression” [26].

### 2.3. Nutritional Status

Nutritional status was evaluated by the nurse using the Mini Nutritional Assessment (MNA^®^), which is a validated screening and assessment tool for older adults in community and hospital settings. The full MNA consists of 18 items (6 questions in the screening part, also called the MNA Short-Form (MNA-SF), and 12 questions in the assessment part) capturing anthropometric measures, dietary intake, appetite, general health, and mobility [5]. The screening part first identifies older adults as “well-nourished” (MNA-SF ≥ 12/14) or “at nutritional risk” (MNA-SF < 12/14), so that the full MNA is performed only if an individual is “at nutritional risk”. The full categorized MNA then classifies individuals into “malnourished” (MNA < 17/30), “at risk of malnutrition” (17/30 ≤ MNA < 24/30) or “well nourished” (MNA ≥ 24/30) [5]. To calculate a full continuous MNA score, individuals with MNA-SF ≥ 12/14 in the screening part were adjusted into a full MNA score (MNA-SF + 16 points) to obtain a full score ranging 0–30 points. Weight and height were measured using standardized scales to the nearest 0.1 kg and 0.01 m, respectively.

### 2.4. Biochemical Measures

Fasting venous blood was collected for the measurement of serum albumin, 25-hydroxyvitamin D (25OHD), parathyroid hormone (PTH), hemoglobin, and C-terminal telopeptide of type 1 collagen (CTx). Serum albumin, and hemoglobin levels were determined using automated standard laboratory methods. Serum 25OHD levels were measured by chemiluminescence immunoassay on a LIAISON^®^ XL analyzer (DiaSorin S.p.A., Saluggia, Italy). Circulating intact PTH was measured by immunochemoluminometric assay performed on ADVIA Centaur^®^ (Siemens Healthcare Diagnostics, Deerfield, MA, USA). Serum CTx levels were measured by electrochemiluminescence immunoassay on a Cobas^®^ 6000 analyzer (Roche Diagnostics International Ltd, Rotkreuz Switzerland). Cut-off values for subnormal levels were 25OHD < 75 nmol/L [27], PTH > 6.9 pmol/L, and hemoglobin <130 g/L (men), <120 g/L (women). Estimated-glomerular filtration rate (eGFR) was calculated from serum creatinine as an indicator of renal function (MDRD formula [28]), with subnormal cut-off values of <60 mL/min/1.73 m^2^. All measurements were performed at the pathology networks affiliated with the Western Health-Sunshine Hospital in St. Albans, Australia.

### 2.5. Post-Fall Care Plan

After review of the results of the complete assessment of the individuals’ risk of falls and fractures by the multi-disciplinary team, individuals were provided with individualized care plans that included pharmacological (e.g., osteoporosis treatment, vitamin D supplements, protein supplements), and non-pharmacological recommendations (e.g., nutrition advice, physical exercise), with a focus on preventing new or recurrent episodes of falls and/or osteoporotic fractures. The care plan was patient-centered through consideration of the risk assessment, individual patient circumstances, and preferences. In consultation with their local general practitioners, individuals were involved in the management of their respective care plan. The current study looks into changes in nutritional status and musculoskeletal components over a period of 6 months.

### 2.6. Musculoskeletal Outcome Measures

Musculoskeletal outcome measures were evaluated by the exercise physiologist as part of standard patient assessment on attendance at baseline and 6-month follow-up. Grip strength (kg) was measured with a handheld JAMAR hydraulic dynamometer (Sammons Preston Inc., Bolingbrook, IL, USA). Individuals had to squeeze the device as hard as possible 3 times in each hand; the highest value was recorded. Gait speed (m/sec) was evaluated using a sensitive walkway (GAITRite system, 16′ model, CIR Systems Inc., Havertown, PA, USA), which recorded spatiotemporal gait speed over 4.8 m with individuals walking at usual speed. The best result of two trials was considered. The Timed Up and Go (TUG) test (sec) measured the time taken to stand up from a standard chair, walk a distance of 3 m, turn, walk back to the chair, and sit down again [29]. The Short Physical Performance Battery (SPPB) is a group of measures that combines the results of the gait speed, chair stand, and balance tests [30]. We included serum CTx levels (assessment described under biochemical measures) as a measure of bone turnover.

### 2.7. Osteopenia/Osteoporosis and Sarcopenia

Body composition and areal bone mineral density (BMD) at three sites (lumbar spine, total hip, and femoral neck) were assessed by the bone densitometrist using a Horizon dual energy X-ray absorptiometry (DXA) machine (Hologic Inc., Bedford, MA, USA). DXA scans were only performed at baseline, and osteopenia/osteoporosis was defined as a BMD T-score <−1.0 SD on at least one of the three regions. As recommended by the Australian and New Zealand Society for Sarcopenia and Frailty Research (ANZSSFR) [31], sarcopenia was defined according to the EWGSOP 2010 definition by fulfillment of low height-adjusted appendicular lean mass (ALM/height^2^) combined with low grip strength or slow gait speed [32]. (ALM/height^2^) was calculated automatically by the DXA machine. We applied the EWGSOP cut-offs for low ALM/height^2^: ≤7.26 kg/m^2^ (♂), ≤5.5 kg/m^2^ (♀); for low grip strength: <30 kg (♂), <20 kg (♀) and for slow gait speed: ≤0.8 m/sec [32]. Osteosarcopenia was defined as the simultaneous presence of osteopenia/osteoporosis and sarcopenia.

### 2.8. Statistical Analysis

Statistical analysis was performed using SAS, version 9.4 (SAS Institute Inc., USA). Statistical significance was based on a two-sided *p*-value <0.05. Normality was assessed using the Shapiro-Wilk test. Mean (standard deviation (SD)) or median (25th percentile (Q1), and 75th percentile (Q3)) were reported for continuous data, and number (percentage (%)) for categorical data. Change (Δ) in continuous variables was calculated as the difference between follow-up and baseline, e.g., ΔMNA = [MNA score (follow-up)—MNA score (baseline)]. To compare baseline and follow-up results, differences were tested using paired *t*-test or Wilcoxon signed-rank test for normally-distributed or not normally-distributed paired samples, respectively.

As proof of concept, multiple linear regression analyses were first performed to test the cross-sectional associations between baseline MNA score and baseline musculoskeletal outcomes. Analyses were adjusted for baseline variables (age, sex, GDS, CACI, and number of medications—all continuous except sex).

For main analysis, individuals were divided into four subgroups based on change in MNA category from baseline to follow-up: (1) Improved nutritional status from baseline to follow-up; (2) Deteriorated; (3) Maintained but remained malnourished or at risk of malnutrition; (4) Maintained and remained well-nourished (reference group). Comparison of clinical, biochemical and musculoskeletal outcome measures between the subgroups vs. the reference group was analyzed using *t*-test or Wilcoxon rank-sum test for normally-distributed or not normally-distributed variables, respectively.

To explore the longitudinal associations between changes in nutritional status and musculoskeletal outcomes, multiple linear regression analyses were performed for each of the musculoskeletal outcome. Change in nutritional status was considered as both continuous (ΔMNA) and categorized exposure. Analyses were adjusted for the baseline outcome, baseline variables (age, sex, GDS, CACI, and number of medications—all continuous except sex), and care plan variables (osteoporosis treatment, vitamin D supplements use, protein supplements use, and physical activity—all categorical). When change in nutritional status was used as continuous exposure (ΔMNA), analyses were additionally adjusted for baseline MNA score. To avoid deletion of information-rich participants, missing values for four binary variables were coded as a separate category. Scatter and residual plots were examined to determine if ΔMNA was related to musculoskeletal changes in a linear manner and if the errors components were independent, homogenous with respect to the variance, and had a mean of zero. If these assumptions were violated, the outcome and/or independent variables were log-transformed to ensure good model fit.

To control for multiple testing, we ranked our hypotheses. Our primary hypothesis is that improvement in nutritional status is associated with greater musculoskeletal recovery. Our secondary hypotheses are that individuals who deteriorated and remained malnourished or at risk of malnutrition are associated with poorer musculoskeletal recovery. As such, *p*-values from multiple linear regression analyses were interpreted in the view of multiple comparisons. If the *p*-value was fairly large (0.01 ≤ *p* < 0.05), we did not interpret them as definitely true, but considered that they may be likely false positive, while very small *p*-values (*p* < 0.01 and *p* < 0.001) were interpreted as likely real findings.

## 3. Results

### 3.1. Descriptive Characteristics, Including Change in Nutritional Status

Out of 254 patients screened at the Falls and Fractures Clinic between October 2016 and December 2018, 106 (76% female) consecutive individuals with median age of 79 (Q1, Q3 72, 82) years were re-assessed at 6-month follow-up. Table 1 presents descriptive characteristics of this study sample. On attendance at baseline, polypharmacy and severe comorbidity were quite prevalent (67% taking ≥5 medications and 45% with a CACI ≥5). The median number of reported falls in the past year was 2 falls. Most individuals (92%) were osteopenic/osteoporotic, and 22% were sarcopenic. On attendance at follow-up, the median number of reported falls in the past 6 months decreased to 0 falls. Moreover, 91 (86%) individuals reported using vitamin D supplements and 5 (5%) protein supplements, 70 (66%) reported having an osteoporosis treatment, and 51 (48%) reported being physically active, as part of the post-fall care plans recommended.

**Figure 1:**
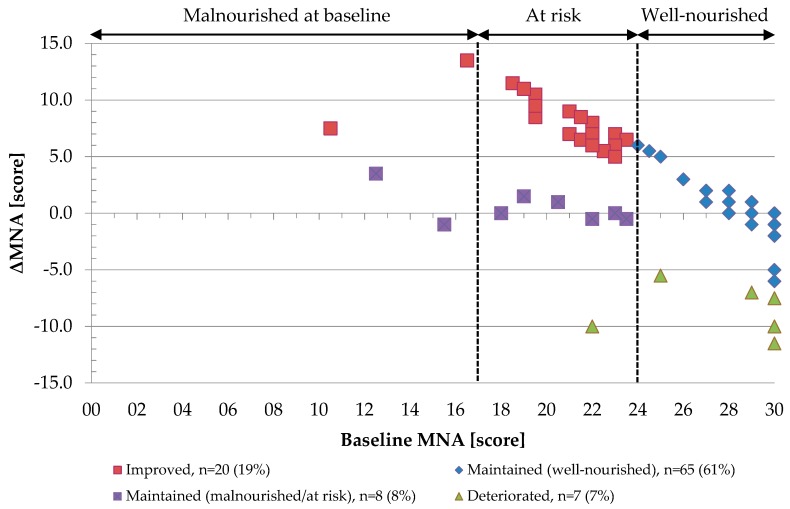
Change in MNA score (ΔMNA) by baseline MNA score across subgroups of nutritional status change. One dot represents one individual. Six participants could not be represented due to missing value in MNA score at follow-up.

The prevalence of malnutrition or risk of malnutrition based on an MNA score <24/30 was 29% at baseline and 15% at 6-month follow-up. Most individuals maintained or improved nutritional status with at least 75% not having a decrease in MNA score (median change of 0.0 (0.0, 3.3) points, *p* = 0.001) (Table 1). Specifically, 73 individuals (69%) maintained nutritional status, including 65 (61%) who remained well-nourished and 8 (8%) who remained malnourished or at risk of malnutrition. Moreover, 20 individuals (19%) improved and 6 individuals (7%) deteriorated nutritional status, as illustrated in Figure 1.

### 3.2. Nutritional Status and Musculoskeletal Health at Baseline

In age-sex adjusted analyses, a 1-point increase in baseline MNA score was associated with an increase of 0.02 m/sec (95% CI 0.00, 0.03, *p* = 0.022) in gait speed and of 0.14 points (95% CI 0.05, 0.23, *p* = 0.003) in SPPB score. Additional adjustment for baseline variables weakened the associations, which became non-significant (Supplementary Table S2).

### 3.3. Changes in Nutritional Status and Musculoskeletal Health

Table 2 compares clinical, biochemical and musculoskeletal measures between subgroups of nutritional status change using the maintained (well-nourished) nutritional status group as reference group. The reference group was associated with significant increase in BMI (*p* = 0.002), weight (*p* = 0.031), 25OHD levels (*p* = 0.013), gait speed (*p* < 0.001), SPPB score (*p* = 0.008) and decrease in CTx levels (*p* < 0.001). Individuals who improved nutritional status were associated with greater increase in BMI (*p* = 0.002) and weight (*p* = 0.006) compared to the reference group, and similar increase in SPPB score (*p* = 0.177). They were not associated with significant improvement in gait speed (*p* = 0.193), but their TUG time significantly decreased (*p* = 0.001). Those who maintained (malnourished/at risk) or deteriorated nutritional status were not linked to significant improvement in any of the variables.

In multiple linear regression analyses (Table 3), change in nutritional status over 6 months showed the strongest associations with SPPB. After adjusting for baseline and care plan covariates, a 1-point increase in MNA score over 6 months was associated with an increase of 0.20 points (95% CI 0.10, 0.31, *p* < 0.001) in SPPB score. In subgroup analyses, individuals who improved nutritional status had for 3.30 sec (95% CI −6.34, −0.26, *p* = 0.033) a larger decrease in time for the TUG test compared to the reference group. Conversely, those who deteriorated in nutritional status had a larger decrease in SPPB score by 1.74 points (95% CI −3.29, −0.20, *p* = 0.028).

## 4. Discussion

Poor nutritional status is subject to intense discussion in geriatric research mainly due to its high prevalence in older adults with falls, associations with higher morbidity and mortality risk, and effects on increased healthcare spending [33]. We assessed change in nutritional status in older adults with a history of falling and how it is related to relevant musculoskeletal changes during post-fall recovery. We found that improvement in nutritional status, based on increase in the MNA score over 6 months, was associated with improvement in physical performance, based on increase in the SPPB score over time.

### 4.1. Changes in Nutritional Status

Approximately one-third (29%) of the studied 106 older adults were malnourished or at risk of malnutrition at baseline. This prevalence is comparable to other studies of community-dwelling older adults using the MNA^®^ (6%–32%) [34]. The prevalence of malnutrition or risk thereof decreased to 15% at follow-up. Comparable observational studies in community-dwelling older adults are lacking, but studies within inpatient settings reported a similar reduction in malnutrition prevalence (10%–13%) based on MNA category change between admission and discharge. However, older adults receiving inpatient services differ significantly in nutritional status and health recovery goals post-discharge to the community, so that results cannot be compared to community-dwelling older adults with confidence [24,35,36].

Most individuals (89%) maintained or improved nutritional status. It is worth noting that 8% of them remained malnourished or at risk of malnutrition at follow-up. A smaller number (7%) deteriorated to an extent sufficient to downgrade MNA category. This implies that while improvement or stabilization of nutritional status is possible during post-fall recovery, a number of individuals may not reach a well-nourished state, despite provision of individualized care plans, which included education and prescription of protein supplements, when indicated. This may have long-term implications for musculoskeletal recovery and quality of life and highlights the need for adequate follow-up of nutritional assessment. Moreover, while it is possible that subtle improvement or deterioration occurred within the stable group, the degree of change may not have been sufficient to alter MNA category.

Our study further demonstrated that MNA change was consistent with significant anthropometric (weight and BMI) changes. This is an important finding, as it is valuable to have a validated nutrition assessment tool to monitor nutrition progress over time, rather than relying only on anthropometric or biochemistry measures such as albumin, which may be confounded by clinical factors such as inflammation [37,38].

### 4.2. Changes in Nutritional Status and Musculoskeletal Health

Over 6 months, there were significant improvements in physical performance (based on gait speed, SPPB score, and TUG test performance) and in CTx levels. For gait speed and SPPB score, improvements were within a range indicating clinically meaningful changes [39], supporting that performance measures may offer a powerful mechanism to act on healthcare needs of older adults at risk for falls.

The observational design of this study prevents us from attributing changes of nutritional status and musculoskeletal outcomes to specific post-fall recommendations or other causes. Care plans were individualized through consideration of patient circumstances and treatment preferences. Incorporation of patients’ decisions about treatment choices and their active involvement in managing their own care plan forms an integrative part of patient-centered medicine [40].

Our research investigated whether changes in nutritional status were reflected by changes in relevant musculoskeletal outcomes post-fall recovery. Improvement in nutritional status, based on a 1-point increase in MNA score over 6 months, was strongly associated with improvement in physical performance, based on an increase of 0.20 points (95% CI 0.10, 0.31, *p* < 0.001) in SPPB score over time. In subgroup analyses, the improved group was significantly associated with decrease in time to perform the TUG test and the deteriorated group with decrease in the SPPB score over time, compared to the reference group. These tools are interrelated and provide valid and reliable measurements of physical performance in community-dwelling older adults, incorporating elements of mobility and balance, with the addition of strength in both the SPPB and TUG test [41]. Nevertheless, caution is warranted when interpreting findings from subgroup analyses, because *p*-values were fairly large (0.01 ≤ *p* < 0.05).

The impact of change in nutritional status on physical performance may be explained by direct or indirect mechanisms. First, increased adequacy of nutritional intake (in terms of quantity and quality) may contribute to recovery of muscle mass and function [42]. This affects physical performance, leading to functional and mobility improvements [10,11]. Improved nutritional status may also be an indicator of decreased comorbidity, which has positive effects on cognitive, functional, and physical performance [8]. Detailed data on changes in disease-related and medical factors could not be considered in this study and may have influenced changes between nutritional status groups and the time taken to recover musculoskeletal health. Finally, there was no association between changes in nutritional status and CTx levels. Physical performance may be more likely than bone turnover to improve alongside nutritional status due to recovery of muscle mass and function.

Our findings support the hypothesis that adequate nutritional follow-up support might increase relevant functional abilities during recovery from a fall. Two recent intervention studies, involving over 200 older adults aged ≥65 years each, showed that nutrition interventions (including enriched diets and/or oral nutritional supplements, home visits and/or telephone follow-ups) yielded significant improvements in weight and functional status over 3 months [43]. Another randomized control study involving over 150 geriatric patients aged >65 years at nutritional risk demonstrated the positive effect of individualized dietician counseling at home after discharge from hospital [44]. Perhaps this study design [44] can be used to conduct larger randomized controlled trials evaluating the effectiveness of specific nutritional interventions and models of care to improve nutritional and musculoskeletal measures in older adults at risk for falls.

### 4.3. Strengths and Limitations

Strengths of the study include the use of validated nutritional and musculoskeletal assessment tools, and the repeated measurements at two time points. The follow-up period of 6 months makes the study appropriate for documenting changes in nutritional status and musculoskeletal outcomes. There are also a number of limitations. The MNA lacks sensitivity to detect subtle changes in nutritional status [36]. As a result, the four nutritional status change subgroups are not evenly represented and are dominated by those who maintained well-nourished nutritional status. Another limitation arises from the selection bias associated with the follow-up design of this study, whereby only those willing to attend a follow-up session were assessed.

A control group was not feasible as routine geriatric care needed to be provided, which included individualized care plans for all patients. This limits the ability to attribute changes observed in musculoskeletal outcomes to specific recommendations. Importantly, it remains unclear whether change in nutritional status has a causal role in change in physical performance, or whether it is a case of reverse causation. The possibility of the temporal association between nutritional status and physical performance being due to residual confounding by unmeasured genetic, lifestyle or environmental factors cannot be ruled out. Finally, our findings may not be generalized because of the heterogeneous and convenience nature of the database.

## 5. Conclusions

Approximately one third of community-dwelling older adults with a history of falling were malnourished or at risk of malnutrition at baseline, and nearly one fifth improved nutritional status at 6-month follow-up. Improvement in nutritional status, based on increase in the MNA score over 6 months, was associated with improvement in physical performance, based on increase in the SPPB score over time. Larger intervention studies are required to ascertain causality and to evaluate specific nutritional interventions and models of care to improve nutritional status and functional recovery in older adults at risk for falls.

## Figures and Tables

**Table 1:** Descriptive characteristics of the study sample, including change in nutritional status.

Characteristic	Descriptive Statistics	All (*n* = 106)
**Baseline**		
Age, year	Median (Q1, Q3)	79 (72, 82)
Female	*n* (%)	80 (75.5)
BMI at baseline, kg/m^2^	Median (Q1, Q3)	27.8 (23.8, 31.9)
Weight at baseline, kg	Median (Q1, Q3)	69.6 (58.5, 85.0)
Height, m	Mean (SD)	1.59 (0.09)
Current smoker ^g^	*n* (%)	11 (10.4)
Number of falls (past 12 months)	Median (Q1, Q3)	2 (1, 2)
Number of fractures (past 5 years)	Median (Q1, Q3)	1 (1, 1)
Severe comorbidity (CACI ≥5)	*n* (%)	48 (45.3)
Polypharmacy (≥5 medications)	*n* (%)	71 (67.0)
Suggestive of depression ^a^ (GDS ≥6/15)	*n* (%)	26 (24.5)
Osteopenia/osteoporosis ^c^	*n* (%)	97 (91.5)
Sarcopenia ^b^	*n* (%)	22 (20.8)
Osteosarcopenia ^b^	*n* (%)	22 (20.8)
***Nutritional status***		
MNA at baseline, score	Median (Q1, Q3)	29 (23, 30)
Malnourished or at risk at baseline (MNA <24/30)	*n* (%)	31 (29.3)
**Follow-up**		
ΔBMI **^h^**, kg/m^2^	Median (Q1, Q3)	0.6 (−0.2, 1.5) *
Δweight **^f^**, kg	Median (Q1, Q3)	0.6 (−1.0, 3.0) *
Number of falls **^d^** (past 6 months)	Median (Q1, Q3)	0 (0, 1)
Number of fractures ^d^ (past 6 months)	Median (Q1, Q3)	0 (0, 0)
Osteoporosis treatment ^e^	*n* (%)	70 (66.0)
Vitamin D supplement use ^e^	*n* (%)	91 (85.9)
Protein supplement use ^e^	*n* (%)	5 (4.7)
Physically active ^j^	*n* (%)	51 (48.1)
***Nutritional status***		
ΔMNA ^d^, score	Median (Q1, Q3)	0 (0, 3.3) *
Malnourished or at risk at follow-up ^d^ (MNA <24/30)	*n* (%)	16 (15.1)

Q1, Q3 = 25th, 75th percentile; CACI = Charlson age-comorbidity index; GDS = Geriatric Depression Scale; MNA= Mini Nutritional Assessment; BMI = body mass index; number of missing values: ^a^ 1, ^b^ 3, ^c^ 5, ^d^ 6, ^e^ 10, ^f^ 14 ^g^ 15, ^h^ 16, ^j^ 27; *: *p* < 0.05 for paired *t*-test or Wilcoxon signed-rank test for the difference between baseline and follow-up values.

**Table 2:** Comparison of clinical, biochemical and musculoskeletal measures, stratified by subgroup of nutritional status change.

Characteristic	Descriptive Statistics	Maintained (Well-Nourished) *n* = 65 (*ref*)	Improved *n* = 20	Maintained (Malnourished/at Risk) *n* = 8	Deteriorated *n* = 7
**Clinical and biochemical**					
Age, year	Median (Q1, Q3)	77 (71, 81)	79 (73, 83)	80 (72, 84)	81 (79, 82)
BMI at baseline, kg/m^2^	Median (Q1, Q3)	29.4 (24.8, 34.2)	25.6 (20.8, 29.6) ^†^	23.8 (18.9, 29.8) ^†^	24.1 (21.4, 27.9) ^†^
ΔBMI ^l^, kg/m^2^	Median (Q1, Q3)	0.4 (−0.3, 1.3) *	1.2 (0.7, 2.5) *^, †^	−0.8 (−2.5, 0.6)	0.0 (−0.3, 1.5)
Weight at baseline, kg	Median (Q1, Q3)	74.0 (64.0, 87.7)	64.1 (51.0, 76.6) ^†^	64.8 (42.3, 70.4) ^†^	64.7 (50.4, 74.0)
Δweight ^k^, kg	Median (Q1, Q3)	0.5 (−0.9, 2.4) *	3.0 (1.0, 5.9) *^,†^	−2.0 (−9.3, 0.0) ^†^	−2.1 (−4.0, 2.8)
Number of falls at follow-up ^f^	Median (Q1, Q3)	0 (0, 0)	0 (0, 1)	1 (0, 2)	2 (0, 2) ^†^
Albumin at baseline, g/L	Median (Q1, Q3)	38.0 (36.0, 40.0)	38.0 (36.5, 40.0)	34.0 (29.5, 39.5)	38.0 (34.0, 41.0)
Δalbumin ^c^, g/L	Median (Q1, Q3)	0.0 (−2.0, 2.0)	0.0 (−2.0, 2.0)	0.5 (−1.5, 4.5)	1.0 (−2.0, 2.0)
25OHD at baseline, nmol/L	Mean (SD)	65.5 (22.5)	74.7 (20.2)	66.0 (23.7)	77.1 (28.3)
Δ25OHD ^a^, nmol/L	Median (Q1, Q3)	4.0 (−7.0, 25.0) *	9.5 (−11.0, 17.5)	8.5 (−5.0, 24.5)	12.0 (−1, 23.0)
PTH at baseline ^f^, pmol/L	Median (Q1, Q3)	6.9 (5.3, 10.3)	7.5 (5.8, 11.2)	6.0 (4.7, 10.5)	5.5 (3.9, 5.7) ^†^
ΔPTH ^g^, pmol/L	Median (Q1, Q3)	−0.1 (−2.0, 2.3)	0.8 (−1.4, 3.8)	1.4 (1.1, 4.9) ^†^	−0.1 (−1.4, 2.6)
Calcium, mmol/L	Mean (SD)	2.4 (0.1)	2.4 (0.1)	2.5 (0.1)	2.5 (0.1)
Phosphate, mmol/L	Mean (SD)	1.2 (0.2)	1.2 (0.2)	1.2 (0.2)	1.2 (0.1)
Hemoglobin at baseline ^a^, g/L	Mean (SD)	130.1 (13.7)	131.5 (13.2)	135.4 (18.9)	130.6 (13.5)
Δhemoglobin ^c^, g/L	Median (Q1, Q3)	1.0 (−4.0, 5.0)	1.0 (−5.0, 5.0)	1.5 (−6.5, 10.0)	5.0 (−11.0, 15.0)
eGFR at baseline, mL/min/1.73 m^2^	Median (Q1, Q3)	75.0 (59.0, 86.0)	67.0 (52.5, 84.5)	85.0 (79.5, 87.5)	59.0 (55.0, 68.0)
ΔeGFR ^d^, mL/min/1.73 m^2^	Median (Q1, Q3)	0.0 (−4.0, 4.0)	−3.0 (−8.0, 1.0)	−3.0 (−13.0, 0.0)	8.0 (0.0, 14.0) ^†^
**Musculoskeletal**					
ALM/height^2^ at baseline, kg/m^2^	Median (Q1, Q3)	6.8 (6.0, 8.1)	6.0 (5.2, 6.8) ^†^	6.2 (5.5, 6.3) ^†^	5.7 (5.3, 6.6) ^†^
Grip strength at baseline ^a^, kg	Median (Q1, Q3)	22.0 (17.0, 28.0)	20.5 (18.0, 26.0)	19.0 (15.0, 24.5)	16.0 (10.0, 24.0)
Δgrip strength ^d^, kg	Median (Q1, Q3)	0.0 (−3.0, 2.0)	0.0 (−2.0, 0.5)	0.5 (−1.5, 1.5)	−3.0 (−4.0, 2.0)
Gait speed at baseline ^e^, m/sec	Median (Q1, Q3)	0.7 (0.5, 1.0)	0.6 (0.5, 0.7)	0.7 (0.5, 0.9)	0.8 (0.6, 0.9)
Δgait speed ^h^, m/sec	Median (Q1, Q3)	0.1 (−0.1, 0.2) *	0.1 (−0.1, 0.2)	−0.0 (−0.1, −0.0) ^†^	−0.1 (−0.2, 0) ^†^
TUG at baseline ^f^, sec	Median (Q1, Q3)	15.2 (10.2, 21.3)	19.4 (16.5, 24.0) ^†^	15.5 (10.9, 19.9)	18.3 (12.6, 22.0)
ΔTUG ^k^, sec	Median (Q1, Q3)	−0.5 (−2.2, 1.3)	−3.2 (−7.4, −0.6) *^,†^	−1.2 (−2.9, 3.4)	−2.6 (−2.9, 1.1)
SPPB at baseline ^b^, score (/12)	Median (Q1, Q3)	7.0 (5.0, 10.0)	6.0 (5.0, 7.0)	6.0 (4.0, 9.0)	7.0 (4.0, 8.0)
ΔSPPB ^g^, score	Median (Q1, Q3)	1.0 (0.0, 2.0) *	1.0 (0.0, 2.0) *	0.0 (−1.5, 1.0)	−1.5 (−2.0, 0.0) ^†^
CTx at baseline ^j^, ng/L	Median (Q1, Q3)	330 (245, 447)	284 (204, 604)	278 (180, 361)	290 (162, 308)
ΔCTx ^m^, ng/L	Median (Q1, Q3)	−127 (−224, −15) *	−116 (−268, −9)	75.0 (−151.0, 102.0)	130 (8, 149) ^†^

SD = standard deviation; Q1, Q3 = 25th, 75th percentile; ALM/height^2^ = height-adjusted appendicular lean mass; BMI = body mass index; 25OHD = 25-hydroxyvitamin D; PTH = parathyroid hormone; EGFR = estimated glomerular filtration rate; TUG test = Timed Up and Go test; SPPB = Short Physical Performance Battery; CTX = C-terminal telopeptide of type 1 collagen; number of missing values: ^a^ 1, ^b^ 2, ^c^ 3, ^d^ 4, ^e^ 5, ^f^ 6, ^g^ 9, ^h^ 11, ^j^ 12, ^k^ 14, ^l^ 16, ^m^ 19; *: *p* < 0.05 for *t*-test or Wilcoxon signed-rank test for the difference between baseline and follow-up values within each group; ^†^: *p* < 0.05 for *t*-test or Wilcoxon rank-sum test for the difference between the improved, maintained (malnourished/at risk) or deteriorated group vs. the maintained (well-nourished) nutritional status group (*ref*), respectively.

**Table 3:** Results of multiple linear regression analyses testing the association between changes in nutritional status and musculoskeletal outcomes.

Change in Musculoskeletal Outcome	Change in Nutritional Status		*n*	Age-Sex Adjusted β (95% CI)	Multivariable Adjusted β (95% CI)
**Δgrip strength, kg**	Categorized ^a^	Improved vs. *ref*	20	−0.17 (−1.98, 1.63)	0.34 (−1.70, 2.73)
		Maintained (malnourished/at risk) vs. *ref*	8	−1.13 (−3.78, 1.52)	−1.22 (−4.07, 1.63)
		Deteriorated vs. *ref*	7	−2.12 (5.00, 0.75)	−1.48 (−4.41, 1.44)
	Continuous ^b^	1-point higher in ΔMNA	96	0.10 (−0.10, 0.30)	0.09 (−0.11, 0.30)
**Δgait speed, m/sec**	Categorized ^a^	Improved vs. *ref*	16	−0.03 (−0.12, 0.07)	0.04 (−0.07, 0.15)
		Maintained (malnourished/at risk) vs. *ref*	8	−0.14 (−0.27, −0.01) ^i^	−0.07 (−0.21, 0.07)
		Deteriorated vs. *ref*	4	−0.15 (−0.33, 0.02)	−0.14 (−0.31, 0.04)
	Continuous ^b^	1-point higher in ΔMNA	89	0.01 (0.00, 0.03) ^i^	0.01 (0.00, 0.02) ^i^
**ΔTUG, sec**	Categorized ^a^	Improved vs. *ref*	17	−3.41 (−5.82, −0.99) ^ii^	−3.30 (−6.34, −0.27) ^i^
		Maintained (malnourished/at risk) vs. *ref*	8	0.04 (-3.25, 3.33)	−0.28 (−4.13, 3.56)
		Deteriorated vs. *ref*	3	−1.87 (−7.09, 3.34)	−1.74 (−7.35, 3.87)
	Continuous ^b^	1-point higher in ΔMNA	86	−0.18 (−0.46, 0.10)	−0.13 (−0.41, 0.15)
**ΔSPPB, score**	Categorized ^a^	Improved vs. *ref*	18	0.40 (−0.54, 1.33)	1.05 (−0.06, 2.15)
		Maintained (malnourished/at risk) vs. *ref*	8	−1.18 (−2.47, 0.11)	−0.72 (−2.13, 0.69)
		Deteriorated vs. *ref*	6	−2.21 (−3.69, −0.72) ^ii^	−1.74 (−3.29, −0.20) ^i^
	Continuous ^b^	1-point higher in ΔMNA	91	0.21 (0.11, 0.31) ^iii^	0.20 (0.10, 0.31) ^iii^
**Δlog (CTx), ng/L**	Categorized ^a^	Improved vs. *ref*	15	0.17 (−0.32, 0.66)	0.00 (−0.56, 0.56)
		Maintained (malnourished/at risk) vs. *ref*	5	0.10 (−0.69, 0.89)	0.10 (−0.79, 1.00)
		Deteriorated vs. *ref*	5	0.60 (−0.19, 1.40)	0.69 (−0.09, 1.48)
	Continuous ^b^	1-point higher in ΔMNA	81	−0.02 (−0.08, 0.04)	−0.04 (−0.09, 0.02)

MNA = Mini Nutritional Assessment; TUG test = Timed Up and Go test; SPPB = Short Physical Performance Battery; CTX = C-terminal telopeptide of type 1 collagen; ^iii^ *p* < 0.001, ^ii^ *p* < 0.01, ^i^ *p* < 0.05: *p*-value of multiple linear regression models testing the associations between changes in nutritional status and musculoskeletal outcomes with the maintained (well-nourished) nutritional status group as reference group (*ref*); ^a^ β coefficient is the change in musculoskeletal outcome associated with change in MNA category from baseline to follow-up. Age-sex model adjusted for the baseline outcome, sex, and age. Multivariable model adjusted for the baseline outcome, baseline variables (sex, age, GDS, CACI, number of medications—all continuous except sex), and care plan variables (osteoporosis treatment, vitamin D supplement use, protein supplement use, physical activity—all categorical); ^b^ β coefficient is the change in musculoskeletal outcome associated with a unit increase in nutritional status over 6 months (ΔMNA). Models also adjusted for baseline MNA score.

## Supplementary Materials

The following are available online at https://www.mdpi.com/2072-6643/11/7/1551/s1, Table S1: The Charlson age-comorbidity index (CACI), Table S2: Cross-sectional associations of baseline nutritional status with baseline musculoskeletal outcomes.

## Abbreviations

25OHD	25-hydroxyvitamin D
ALM	Appendicular lean mass
ANZSSFR	Australian and New Zealand Society for Sarcopenia and Frailty Research
BMD	Bone mineral density
BMI	Body mass index
CACI	Charlson age-comorbidity index
CI	Confidence interval
CTx	C-terminal telopeptide of type 1 collagen
DXA	Dual energy X-ray absorptiometry
EWGSOP	European Working Group on Sarcopenia in Older People
eGFR	Estimated glomerular filtration rate
GDS	Geriatric Depression Scale
MNA	Mini Nutritional Assessment
PTH	Parathyroid hormone
Q1	25th percentile
Q3	75th percentile
SD	Standard deviation
SPPB	Short Physical Performance Battery
TUG test	Timed Up and Go test
