# Supplementary material for: Changes in Nutritional Status and Musculoskeletal Health in a Geriatric Post-Fall Care Plan Setting
Source: Nutrients. 2019 Jul 9;11(7):1551. doi: 10.3390/nu11071551 (PMC6682897; doi:10.3390/nu11071551)
Supplement: Supplementary file 1 [file nutrients-11-01551-s001.pdf]

Table S1. The Charlson age-comorbidity index (CACI).

#	Comorbid condition	Weight
1	Myocardial infarction	
2	Congestive heart failure	
3	Peripheral vascular disease	
4	Cerebrovascular disease	
5	Chronic pulmonary disease	1
6	Connective tissue disease	
7	Ulcer disease	
8	Mild liver disease	
9	Diabetes	
10	Hemiplegia	
11	Moderate/severe renal disease	
12	Diabetes with end-organ damage	2
13	Any tumor	
14	Leukemia	
15	Lymphoma	
16	Moderate/severe liver disease	3
17	Metastatic solid tumor	6
18	AIDS	
Age	For each decade over age 40 years	1

AIDS= Acquired immune deficiency syndrome; adapted from Charlson et al.[1]

The Charlson comorbidity index (CCI) is a tool that was originally developed to facilitate classification of comorbidities in longitudinal studies [2]. Seven years following the publication of their original work, Charlson et al. proposed that a modified, combined age-comorbidity index (Charlson age-comorbidity index (CACI)) could be helpful in smaller studies with a more limited number of outcome events [1]. The CACI, whose validity and reliability has been demonstrated in various geriatric patients, combines 19 medical conditions, weighted 1 to 6, with age weighted 1 for every decade past 40 years old (Table S1).

References

1. Charlson, M.; Szatrowski, T.P.; Peterson, J.; Gold, J. Validation of a combined comorbidity index. *J Clin Epidemiol* **1994**, *47*, 1245-1251.
2. Charlson, M.E.; Pompei, P.; Ales, K.L.; MacKenzie, C.R. A new method of classifying prognostic comorbidity in longitudinal studies: development and validation. *J Chronic Dis* **1987**, *40*, 373-383.

Table S2. Cross-sectional associations of baseline nutritional status with baseline musculoskeletal outcomes.

Baseline Musculoskeletal Outcome	Baseline Nutritional Status		*n*	Age-Sex Adjusted β (95% CI)	Multivariable Adjusted β (95% CI)
**Grip strength, kg**	Continuous [b]	1-point higher in MNA score	104	0.23 (-0.05, 0.52)	-0.02 (-0.21, 0.27)
**Gait speed, m/sec**			100	0.02 (0.00, 0.03) [i]	-0.00 (-0.02, 0.01)
**log (TUG), sec**			98	-0.01 (-0.03, 0.01)	0.01 (-0.01, 0.03)
**SPPB, score**			103	0.14 (0.05, 0.23) [ii]	0.00 (-0.09, 0.10)
**log (CTx), ng/L**			93	-0.01 (-0.04, 0.02)	0.00 (-0.03, 0.04)

MNA= Mini Nutritional Assessment; TUG test= Timed Up and Go test; SPPB= Short Physical Performance Battery; CTX= C-terminal telopeptide of type 1 collagen; [iii]: $p<0.001$, [ii] $p<0.01$, [i] $p<0.05$ for multiple linear regression models testing the associations between baseline MNA score with baseline musculoskeletal outcomes; [b] ß coefficient is the 1-unit increase in baseline musculoskeletal outcome associated with a 1-point increase in baseline MNA score. Age-sex model adjusted for age and sex. Multivariable model adjusted for baseline variables (age, sex, GDS, CACI, number of medications – all continuous except sex).
